# Physicians without Chests: Character Formation in Medical School

**DOI:** 10.1007/s40670-026-02837-x

**Published:** 2026-07-25

**Authors:** Daniel J. Hurst, Anne Jones, Christopher A. Bobier, Jennifer Fischer

**Affiliations:** 1 Department of Medical Education and Scholarship, Rowan-Virtua School of Osteopathic Medicine, Stratford, NJ USA; 2 Department of Family Medicine, Rowan-Virtua School of Osteopathic Medicine, Stratford, NJ USA; 3 College of Medicine, Central Michigan University, Mt. Pleasant, MI USA

**Keywords:** Character, Ethics, Medical, Education, Medical, Undergraduate, Professionalism

## Abstract

Modern medical education risks producing, in the words of C.S. Lewis, “men without chests”—individuals who possess the cognitive “head” to analyze ethics and the “belly” of technical appetites, but lack the “chest”: the stable moral sentiments and virtues required to bridge knowledge and action. Drawing on Aristotelian ethics and Lewis’ insights, this paper argues that prioritizing cognitive mastery and performative professionalism is insufficient for true character formation. Despite the rise of Professional Identity Formation, the hidden curriculum—which often values efficiency over empathy—may undermine formal instruction, leading to situations where students select correct answers on exams without internalizing the virtues necessary for clinical practice. To bridge this gap, medical education must shift from mere information to intentional formation. We propose a systemic realignment across three domains: (i) admissions, (ii) assessment, and (iii) institutional culture. By embracing education as the cultivation of the “chest,” the profession can move beyond producing technically competent practitioners to forming virtuous physicians grounded in practical wisdom.

## Introduction

Over two millennia ago, Aristotle argued that the ultimate aim of human life is *eudaimonia*—a state of flourishing achieved through the cultivation and exercise of virtue purposefully developed over a lifetime [1]. Virtues, on this account, are not innate traits but acquired dispositions, formed through thoughtful habituation and refined through practice, that constitute good character. They shape not only what one does, but how one perceives, reacts, judges, and feels. Education, for Aristotle, is not merely the transmission of knowledge but also the formation of character—equipping individuals with the practical wisdom (*phronesis*) necessary to navigate complex and uncertain circumstances virtuously or well.

This classical insight has seen a modest resurgence in contemporary higher education. Character education initiatives–particularly at the undergraduate level–have sought to embrace the formative dimensions of education, emphasizing virtue, purpose, and moral development alongside intellectual achievement [2, 3]. Yet medical education, despite its explicitly moral aims (e.g., Edmund Pellegrino’s refrain: “Medicine is at heart a moral enterprise.” [4]), has been slower to follow suit. If any profession requires not only technical competence but also well-formed character, it is medicine.

The shift to scientific-based teaching and away from humanistic education may be an unintended consequence of the 1910 Flexner Report. In its pursuit of professionalizing medicine and establishing rigorous scientific standards, the Report inadvertently codified a dualism that persists today. By grounding the curriculum in laboratory science it prioritized the scientist-clinician, and while this protected public safety through standardized knowledge, it also relegated the “art” of medicine—including character development and moral philosophy—to the periphery of formal education [5]. In addition, medical admissions standards pushed towards a stronger foundation in scientific disciplines which then reduced the amount of time available for college graduates to devote to humanities prior to entering medical school [6].

At medical schools in the United States, professionalism and medical ethics are the domains most closely associated with character formation. Students are introduced to ethical frameworks, taught the principles of autonomy, beneficence, nonmaleficence, and justice, and assessed on their ability to apply these concepts in standardized scenarios. These domains are also represented—albeit unevenly—on licensing examinations. But here lies the problem: the ability to select the correct answer on an exam is a poor proxy for the possession of virtue or character. One may competently analyze a case involving informed consent while lacking the honesty or courage required to navigate the complexity of these situations in practice [7]. What emerges is a subtle but consequential shift: ethics education becomes oriented toward cognitive mastery rather than character formation. Students learn to think about ethics without necessarily being formed to live it.

The empirical record supports this concern. Disciplinary action of physicians by a medical licensing board has been strongly associated with prior unprofessional behavior during medical school [8, 9], potentially indicating that initial professionalism concerns are not incidental lapses, but rather symptomatic of deep-seated habits of character. Additional studies have documented erosion in empathy during medical training, particularly during the clinical years, alongside increasing cynicism and moral distress. One frequently cited longitudinal study found that medical students’ empathy scores decline significantly over time [10], raising concerns about the formative impact of clinical environments. Taken together, these considerations suggest that medical education does not merely transmit knowledge; it shapes who we are, for better or for worse.

Yet even where ethics and professionalism are formally taught, the broader educational ecosystem may be shaping students in countervailing ways. The so-called “hidden curriculum”—the set of implicit values conveyed through institutional culture, role modeling, and systemic pressures—may exert a more powerful formative influence than formal instruction. What emerges is a divergence between the individual’s ability to identify moral principles and their increasing adoption of practices that minimize interpersonal empathy and adherence to professional ethics.

## Professional Identity Formation

In recent years, medical education has increasingly turned to the concept of professional identity formation (PIF) as a way of addressing precisely this gap between knowledge and practice. Rather than conceiving professionalism as a set of discrete behaviors or competencies, PIF frames medical education as a process through which learners come to “think, act, and feel like physicians [11].” On this account, the goal is not merely that students perform professional behaviors, but that they internalize the values, norms, and dispositions that constitute the professional role. This shift represents an important conceptual advance. It acknowledges that ethical practice cannot be reduced to the application of abstract principles, nor to the demonstration of isolated skills. Instead, it emerges from the gradual integration of knowledge, values, and experience into a coherent sense of self. Professional identity is thus formed through processes of socialization—through participation in clinical environments, interaction with patients and mentors, and engagement with the moral challenges inherent to medical practice [12].

The literature on PIF underscores the central role of the learning environment in shaping this process. Formal curricula play a role, but they are neither primary nor sufficient. Rather, identity formation is profoundly influenced by informal and hidden curricula: the behaviors modeled by faculty, the norms reinforced by institutional structures, and the implicit messages conveyed about what is valued in practice [13]. Structured reflection, mentorship, and narrative practices have therefore been emphasized as mechanisms for helping students make sense of formative experiences and integrate them into their developing professional identities [14].

Despite its promise, however, the PIF framework faces several limitations. First, it is often invoked descriptively rather than operationalized pedagogically. Second, and more importantly, PIF can be undermined by the very environments in which it unfolds. If the hidden curriculum prioritizes efficiency over presence, hierarchy over moral courage, or detachment over empathy, then the identities formed may reflect these priorities rather than the profession’s stated ideals. In this sense, PIF does not automatically produce virtuous physicians; it may produce physicians whose identities are shaped by the dominant values of their particular training context, which would vary by location, site, specialty, or system.

Finally, the relationship between PIF and competency-based medical education (CBME) remains unsettled. While CBME has sought to define and assess professional behaviors with increasing precision, critics have noted that competence alone is insufficient [15]. One can demonstrate professionalism in isolated contexts without possessing a stable professional identity oriented toward the good of patients. Conversely, identity formation cannot be reduced to a checklist of observable behaviors. The risk, therefore, is that professionalism—and by extension PIF—becomes performative; something learners learn to display in evaluative settings without internalizing its underlying moral commitments.

### Physicians without Chests

It is here that the diagnosis offered by C. S. Lewis in *The Abolition of Man* becomes particularly illuminating [16]. Lewis lamented back in 1943 that modern education risks producing “men without chests”—individuals whose rational capacities (the “head”) and appetites (the “belly”) remain intact, but whose moral sentiments (the “chest”) are underdeveloped or malformed. While the language reflects its historical context, the underlying concept remains salient: the chest denotes the seat of rightly ordered affections—the trained dispositions (i.e., virtues) that enable one to respond appropriately to value. It is what links knowledge of the good to action, reason to rightly ordered desire.

Without this mediating refinement of affection, moral knowledge becomes inert. One may recognize that a patient deserves dignity without feeling the weight of that claim, and therefore without acting upon it. One may know in the abstract that a particular patient should be treated with compassion but still act from frustration or indifference. In this sense, the person “without a chest” is not ignorant of what is the good or the right thing to do; they are insufficiently formed to be moved by it.

Contemporary medical education, for all its strength in clinical reasoning, risks producing this kind of practitioner: physicians without chests. Students are trained in the cognitive dimensions of ethics: identifying principles, analyzing dilemmas, and selecting appropriate responses in controlled settings. Yet far less attention is given to the cultivation of the moral virtues (e.g., practical wisdom, compassion, respect) that enable ethical action under real-world conditions—conditions often marked by time pressure, hierarchy, uncertainty, and competing obligations.

Indeed, elements of the hidden curriculum may actively undermine such formation. Efficiency is often prioritized over presence, subtly discouraging the kind of attentiveness required for compassionate care. Hierarchical structures may inhibit moral courage, conditioning trainees to remain silent in the face of questionable practices. Emotional detachment, while sometimes necessary, can be misinterpreted as professionalism and thereby reinforced. Over time, these pressures do not merely constrain behavior; they shape character in ways potentially undetected by the individual, and thus the profession itself.

From the institutional perspective, it has been proposed that the hidden curriculum has stunted the enhancement of professionalism as a competency in order to preserve the elitist status of the profession itself [17]. By failing to connect the loose threads between ethical principles, virtues, and habit formation through trial and error, the reduction of professionalism to a set of performative checklists maintains the sense of mystery around what makes a good doctor, and perpetuates the production of “physicians without chests” who then submit to the hierarchy and authoritarian structure of medicine. When these physicians eventually encounter the realities of clinical practice, they may find themselves in a state of moral crisis. Having the “head” to recognize a moral dilemma but lacking the “chest” to navigate it, they may fall into moral distress, finding that their actions have become inconsistent with their deepest values, leaving a wound where their character should have been formed [18].

The result is a disjunction: physicians who can articulate ethical principles but struggle to embody them consistently in practice. In Lewis’ terms, we “make men [physicians] without chests and expect from them virtue and enterprise.” We expect physicians to act with integrity, courage, and compassion in morally fraught situations, yet we have not adequately formed them to do so.

## What Can be Done?

If this diagnosis is correct, reform must move beyond simply adding more ethics and professionalism content to an already crowded curriculum. The issue may not primarily be one of *quantity* but of *orientation*. If medical education is to address the gap between ethical knowledge and ethical action, it must embrace an account of education as formation.

One promising avenue lies in CBME [19]. At least in principle, CBME offers a conceptual opening for rethinking ethics and professionalism not as domains of knowledge acquisition, but as sets of observable, assessable behaviors cultivated over time. Yet, CBME relies heavily on having seasoned faculty who are able to guide and mentor students along through the competencies. Faculty must be fully committed to this educational mission for it to succeed. Furthermore, without integration with PIF, CBME risks reducing professionalism to a checklist rather than cultivating enduring dispositions. A more comprehensive approach would treat ethical practice as both performative and formative: behaviors are not merely indicators of competence but sites of habituation through which character is formed.

This has implications for assessment. Rather than relying primarily on multiple-choice examinations, medical education should increasingly evaluate ethical capacities in context—through standardized patient encounters, workplace-based assessments, and multisource feedback. Such approaches better capture the patterns of action through which professional identity is constituted. At the same time, current efforts to assess “character” at the point of admission illustrate the limitations of remaining within a primarily cognitive paradigm. Situational judgment tests such as Casper, a computer-based admissions assessment used by many medical schools to evaluate interpersonal and professional attributes through responses to hypothetical scenarios, aim to evaluate applicants’ ethical reasoning. While these instruments acknowledge the insufficiency of traditional academic metrics, the drawback is that they may largely reproduce the assumption that moral capacity can be inferred from decontextualized responses to hypothetical dilemmas [20]. They risk conflating the articulation of ethical reasoning with the possession of stable moral dispositions.

Insights from character education development in postgraduate programs further clarify what is missing. Recent interdisciplinary work by Lamb and colleagues, drawing on philosophy, psychology, and education, suggests that virtue is cultivated not primarily through the acquisition of ethical knowledge but through sustained educational practices that shape stable moral dispositions over time. Their Aristotelian model of character formation emphasizes that character develops through multiple, mutually reinforcing educational strategies. Seven complementary strategies for cultivating virtue are identified: habituation through repeated practice, structured reflection on personal experience, engagement with virtuous exemplars, dialogue that increases moral and virtue literacy, awareness of situational variables that shape behavior, the use of moral reminders to reinforce commitments, and the development of relationships characterized by mutual accountability [21]. Taken together, these approaches emphasize that character is not formed through isolated instruction but through sustained, integrated engagement across contexts. They align closely with the aims of PIF, while offering a more explicit account of the mechanisms by which stable dispositions are cultivated over time.

## A Call to Action: From Information to Formation

To move beyond producing “physicians without chests,” medical education must transition from mere cognitive ethics and performative professionalism to include character formation. We recognize that assessing character is substantially more challenging than assessing knowledge or many clinical skills. Character cannot be reduced to a single instrument, checklist, or isolated observation, nor should high-stakes decisions rest on individual episodes of perceived unprofessionalism. Rather, meaningful assessment requires the longitudinal synthesis of multiple sources of evidence—including direct observation, narrative assessments, multisource feedback, patterns of behavior over time, and learners’ responsiveness to feedback. The goal is not to measure virtue directly but to evaluate the developmental trajectory of professional formation. While we don’t underestimate the need for additional research and a deeper evidence base to inform these recommendations, we preliminarily suggest that this diagnosis may require a systemic realignment across three key domains:

1. Admissions: Screening for growth potential

Rather than relying on decontextualized situational judgment tests, admissions should identify applicants with a high propensity for character development rather than attempting to identify those presumed to possess fully formed virtue.

- Predictive Traits: Prioritize characteristics such as responsibility, intellectual humility, and a willingness to seek, receive, and incorporate feedback. Research suggests (and our experience concurs) that these characteristics are predictive in determining who has the propensity for further character development in medical school [8].
- Targeted Inquiry: Use interview questions that explore applicants’ established habits, core values, responses to failure, and past experiences navigating ethical failure, character testing, or receiving difficult feedback. The goal should be to identify coachability and a demonstrated commitment to personal growth rather than polished responses to hypothetical scenarios.

2. Longitudinal Curriculum with Assessment

Character is not a discrete competency to be checked off, but a set of stable dispositions cultivated through repeated practice, reflection, feedback, and experience over a lifetime, and, accordingly, character formation should be embedded longitudinally throughout undergraduate medical education rather than confined to isolated professionalism sessions or ethics courses. In a CBME model, assessment of competencies is an ongoing process that begins in the pre-clerkship curriculum, is shaped by faculty mentors, and aims toward a continuous process of feedback and the learner utilizing that feedback to improve their performance.

- Skill Integration and Longitudinal Monitoring: Longitudinally integrate ethics and professionalism into pre-clerkship and clerkship training not as ancillary but as integral to the curriculum. Implement tracking systems to detect and remediate unprofessional habits early, preventing unvirtuous behaviors from becoming deep-seated, as well as acknowledging good habits in order to reinforce the virtues that should be continued.
- Connecting Behaviors: Explicitly link classroom habits, such as timeliness and deadline adherence, to clinical outcomes like documentation accuracy and patient safety.
- Situational Awareness and Critical Ethical Reasoning: Move away from “right answer” multiple-choice formats toward developing situational awareness and critical ethical reasoning for real-world dilemmas.
- Structured Reflection: Incorporate recurring opportunities for guided reflection following ethically challenging clinical experiences, helping learners connect lived experience with ethical reasoning and cultivate practical wisdom.
- Direct Observation and Developmental Feedback: Utilize direct observation of clinical care and multi-source (360-degree) feedback to evaluate professional conduct in real-time.
- Transcript Recognition: Hold behavior to account on the official transcript, treating professional conduct as a longitudinal domain of competence deserving the same educational attention and institutional weight as clinical knowledge.

3. Institutional Culture: Modeling and alignment

Character formation is ultimately undermined if the hidden curriculum rewards efficiency over empathy, productivity over integrity, or technical excellence over moral excellence. Educational institutions must therefore ensure that the values they profess are reflected consistently in their culture. At the same time, institutions must recognize that not every lapse in professionalism reflects a grave deficiency of character. Burnout, depression, anxiety, moral distress, and other forms of psychological suffering may manifest as diminished empathy, withdrawal, irritability, or apparent unprofessionalism. Educational cultures that distinguish between misconduct, willful or unintentional, and learners in distress are better positioned to respond with both accountability and appropriate support. Such an approach models the very virtues of compassion, justice, and practical wisdom that institutions seek to cultivate.

- Growth Mindset: Faculty, staff, and administration must model growth mindsets and openness to feedback, creating the psychological safety necessary for students to admit and learn from mistakes [22].
- Institutional Self-Assessment: Schools should use rubrics to audit their own values across all operations—from research to patient care—ensuring institutional integrity matches their educational aims.
- Transparency: Embrace remediation and grace, allowing students to experience the trial and suffering required for true character strengthening, which is emblematic of a just culture [23].

## Conclusion

Taken together, these approaches suggest that the central task of medical education is not merely to teach ethical principles or even to assess professional behaviors, but to form professional identities grounded in virtue. In the language of C. S. Lewis, this is nothing less than the cultivation of the “chest”—the development of rightly ordered moral sentiments that connect knowledge to action. Without this, both competency frameworks and identity-based approaches risk producing clinicians who can recite ethical principles without being truly formed by them.

## Data Availability

Not applicable.
